# Tracheotomy

**Published:** 1832

**Authors:** 


28. *Tracheotomy.* By Dr. Wells.—On the 16th of September, a child, four years of age, suffered a water-melon seed to pass into the trachea. Six days after the accident he was brought to Dr. Wells, who found that the breathing had become permanently difficult and croupy, the intervals between the convulsive paroxysms short, the face livid and the pulse small and too frequent to be numbered.

The operation was decided upon immediately, and the patient having been placed in a proper position, an incision was made along the median line, from a little below the cricoid cartilage to the upper end of the sternum, a vein of considerable size, which was divided and bled profusely, was secured by a ligature. The tracheal rings were divided to the extent of three-fourths of an inch, and the parts held asunder by two slender instruments. The air and bloody foam rushed from the opening with great violence, and the seed was thrown out over the head of one of the attendants, to the distance of three yards. The wound was closed by sutures and adhesive straps, and irritation allayed by anodynes combined with antimonials. Notwithstanding the dressings, the air continued to pass by the wound for three or four days, after which the wound soon healed. The operation was followed by no other inconvenience than a temporary hoarseness.

Dr. W, thinks the operation should always be attempted, provided the offending body continues moveable in the trachea, a state of things indicated by paroxysms of difficult breathing. He also thinks laryngotomy preferable to tracheotomy.

This operation has been lately performed with a degree of skill that merited a favorable termination, by Dr. McCulloch, of Mount Pleasant, in the vicinity of this city. The patient, a child about two years of age, was playing upon his back with some grains of corn in his mouth, when one of them slipped into the trachea. The violent paroxysms of convulsive breathing occurring at short intervals, induced the parents to submit the child to the operation, and it was accordingly performed on the second day. Laryngotomy was performed without difficulty, no hæmorrhage occurring to impede the operation, but the foreign body could not be found. The patient lived six days after the operation, and after death, the corn was found in the trachea imbedded in mucus.

